# Massive hemothorax induced by pulmonary arteriovenous malformation rupture: a case report and literature review

**DOI:** 10.1186/s13019-024-02867-9

**Published:** 2024-06-21

**Authors:** Xiang Li, Lijun Duan, Shengnan Mu, Xin Dong, Xiaoqian Lu, Dianbo Cao

**Affiliations:** Department of Radiology, the First Hospital of Jilin University, No. 71 of Xinmin Street, Changchun, Jilin 130021 China

**Keywords:** PAVM, HHT, Hemothorax, CT angiography, Treatment

## Abstract

**Background:**

Pulmonary arteriovenous malformation (PAVM), also known as pulmonary arteriovenous fistula, is a rare vascular developmental anomaly. Most cases of PAVM are associated with hereditary hemorrhagic telangiectasia (HHT). Hemothorax associated with PAVM is even rarer, and management concerning this complication still challenges.

**Case presentation:**

A 55-year-old man with sudden onset of dyspnea and chest pain was admitted to our hospital. He had a medical history of epistaxis, intraperitoneal germ cell tumor and PAVM. Chest unenhanced CT revealed the left-sided pleural effusion together with partial passive atelectasis and gradual increase at the interval of six days. Diagnostic thoracocentesis further revealed hemorrhagic effusion. CT angiography (CTA) showed tortuously dilated lumen of the left lower pulmonary artery and PAVM with the formation of aneurysm. Due to his family's refusal of surgery, the patient underwent transcatheter embolization therapy. However, the left pleural effusion did not significantly reduce and there was a slow drop in hemoglobin value even after interventional treatment, indicating the possibility of ongoing active bleeding. Eventually, the patient received lobectomy of the left lower lobe with a satisfactory outcome.

**Conclusions:**

Massive hemothorax resulting from PAVM rupture into the pleural space can lead to fatal outcomes. CTA can accurately diagnose this pathologic condition. Transcatheter embolization is frequently used in the treatment of PAVM, but it may be challenging to achieve the desirable effect in patients with hemothorax. Combined with our case and literature review, direct radical surgery can lead to a successful outcome when PAVM complicated with hemothorax and a large diameter of the draining vein.

## Introduction

Pulmonary arteriovenous malformation (PAVM) is a rare, low-resistance, high-flow abnormal vascular structures that connect a pulmonary artery to a pulmonary vein, bypassing the normal pulmonary capillary bed and resulting in an intrapulmonary right-to-left shunt [1]. Most PAVMs are congenital and closely associated with hereditary haemorrhagic telangiectasia (HHT), also known as Osler-Weber-Rendu syndrome. It is an autosomal dominant inherited disease [2]. Most PAVMs do not cause symptoms, but a few may lead to chest pain, breathlessness, cyanosis and paradoxical embolism.

Spontaneous hemothorax secondary to PAVM rupture is an uncommon complication that can be life-threatening if not promptly diagnosed and treated [3, 4]. Therefore, once diagnosed, it is of vital importance to apply appropriate treatment strategies [5]. For routine PAVM cases without complications and with smaller drainage vein, interventional embolization is widely used in clinical practice. However, in some regions transcatheter embolization is still considered as the preferred treatment for PAVM, even complicated with hemothorax and pulmonary hematoma. Based on the literature review and our clinical practice, this approach does not guarantee success for all cases. Therefore, individualized treatment must be fully taken into account for achieving the goal of precision medicine.

Here, we present a case of a giant PAVM complicated by hemothorax and perform a PubMed search to review 30 similar cases from January 2000 to July 2023. The reasons for the initial failure of interventional embolization are discussed through comprehensive evaluation based on our case and literature review. By studying and analyzing previous cases, we try to summarize the evidence regarding consideration for the diagnosis and treatment of PAVM complicated with hemothorax.

## Case presentation

**Fig. 1:**
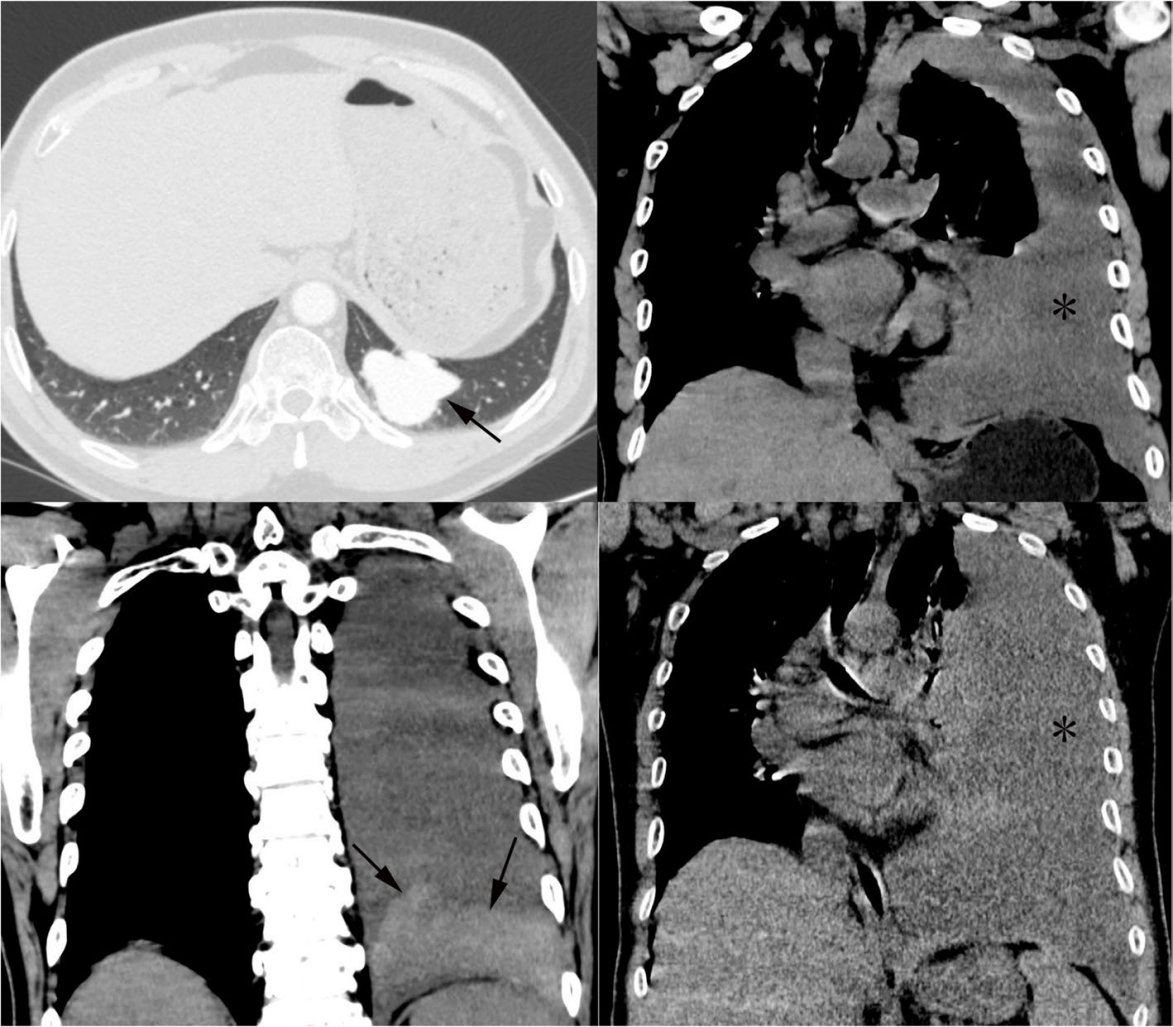
**A** Contrast-enhanced CT revealed PAVM with the formation of aneurysm 18 months previously. **B**-**C** Chest unenhanced CT indicated the left pleural effusion together with irregular patchy high-dense opacity at the upper aspect of the diaphragm. **D** Chest CT at the interval of six days showed a prominent increase in the amount of left pleural effusion

**Fig. 2:**
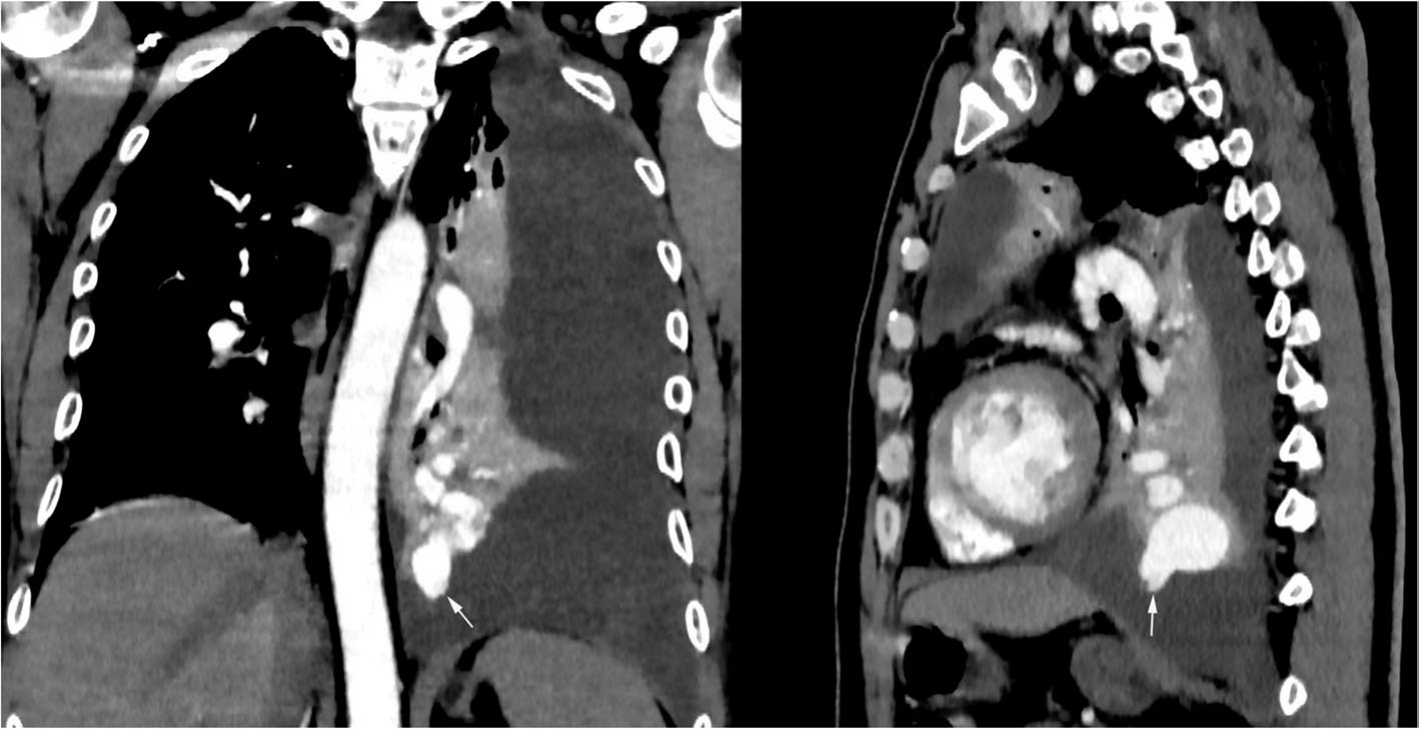
**A**-**B** Pulmonary arterial CT angiography demonstrated an irregularly enhanced mass communicating with the tortuous pulmonary vessels in the left lower lobe

**Fig. 3:**
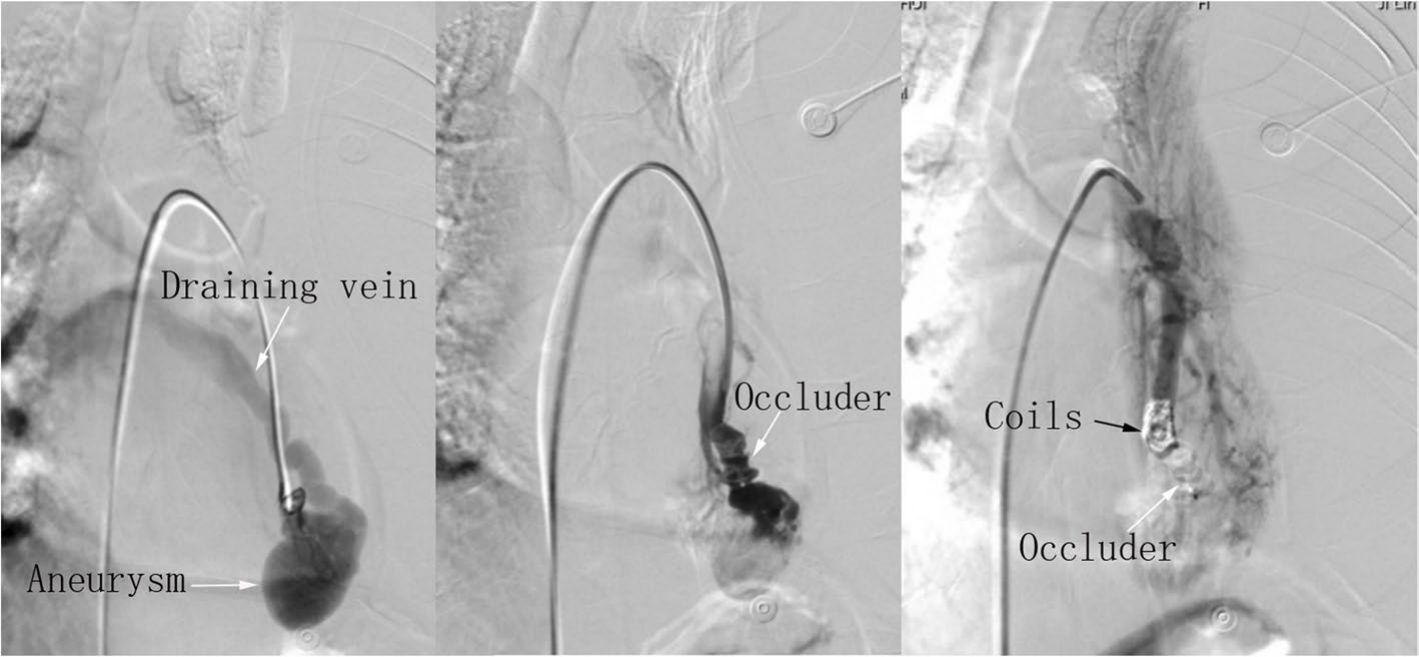
**A** Digital subtraction angiography showed typical PAVM with the formation of pulmonary aneurysm, with a larger draining vein. **B** DSA showed an unsatisfactory embolization after the deployment of Amplatzer septal occluder alone. **C** Amplatzer septal occluder concomitant with six steel coils resulted in the aneurysmal disappearance of the PAVM on the repeated DSA

A 55-year-old man was admitted with sudden left-sided pleuritic chest pain and dyspnea after a fight. He had a medical history of epistaxis and intraperitoneal germ cell tumor, but no history of any chest trauma. Previous chest CT revealed a PAVM, but it did not rupture then (Fig. 1A). On physical examination, his vital signs were normal, but there had been a recent low-grade fever, with a temperature of around 37.5℃. There were decreased breath sounds in the left lower lung, dullness in percussion sound, and telangiectases in oral mucosa and fingertips. Laboratory blood tests showed mild anemia (hemoglobin 95 g/L and hematocrit 29.7%). Chest unenhanced CT revealed the presence of left pleural effusion together with irregular patchy high-dense opacity (Fig. 1B-C), which indicated the possibility of PAVM rupture with bleeding. Follow-up CT showed a remarked increase in the amount of left pleural effusion at the interval of six days (Fig. 1D). Diagnostic thoracocentesis revealed hemorrhagic exudate. Pulmonary arterial CT angiography (CTA) revealed an irregularly enhanced mass with communication between pulmonary artery and vein in the left lower lobe of the lung, measuring approximately 3.9 cm × 3.1 cm. These manifestations were strongly suggestive of a PAVM and the formation of aneurysm. Meanwhile, the irregular shape of PAVM strongly suggested rupture of PAVM (Fig. 2A-B). After multidisciplinary consultations, surgical treatment was recommended as the first choice. His family refused surgery and opted for interventional treatment. Under local anesthesia, the embolization procedure was established through the right femoral vein on 28 February, 2022. Firstly, an 8 French sheath, 8 French guiding catheter, and 5 French pigtail catheter were inserted. Then, a 260 cm guide-wire was introduced to guide them heading for the left pulmonary artery, ultimately reaching the vicinity of PAVM. Digital subtraction angiography (DSA) showed typical PAVM with the formation of pulmonary aneurysm lacking contrast extravasation (Fig. 3A). Due to the relatively large diameter of the patient’s feeding artery, Amplatzer septal occluder resulted in unsatisfactory embolization (Fig. 3B). Subsequently, six steel coils were deployed, and repeated DSA revealed the aneurysmal disappearance of the PAVM (Fig. 3C).

**Fig. 4:**
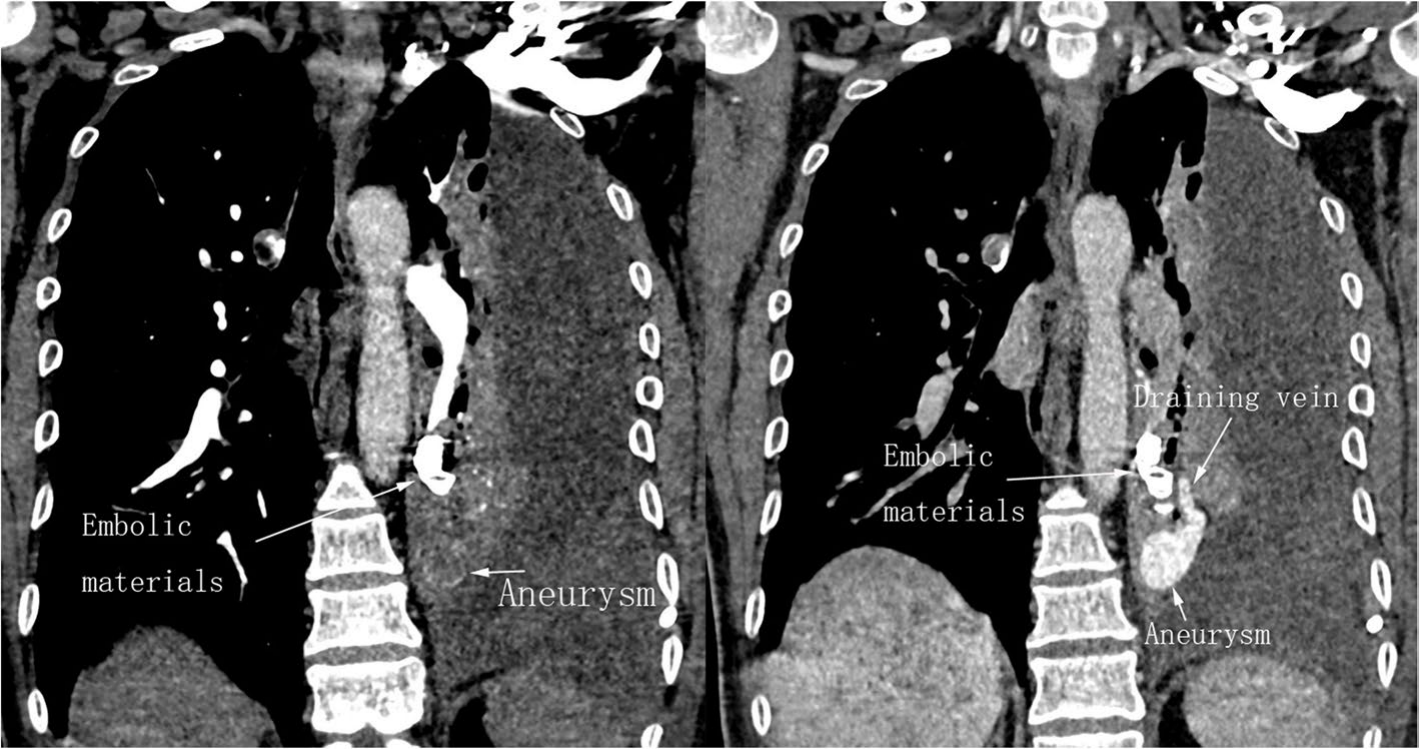
**A**-**B** Follow-up contrast-enhanced CT showed a large amount of fluid accumulation in the left pleural cavity, partial lung collapse and remnant of arteriovenous fistula in the delayed phase

**Fig. 5:**
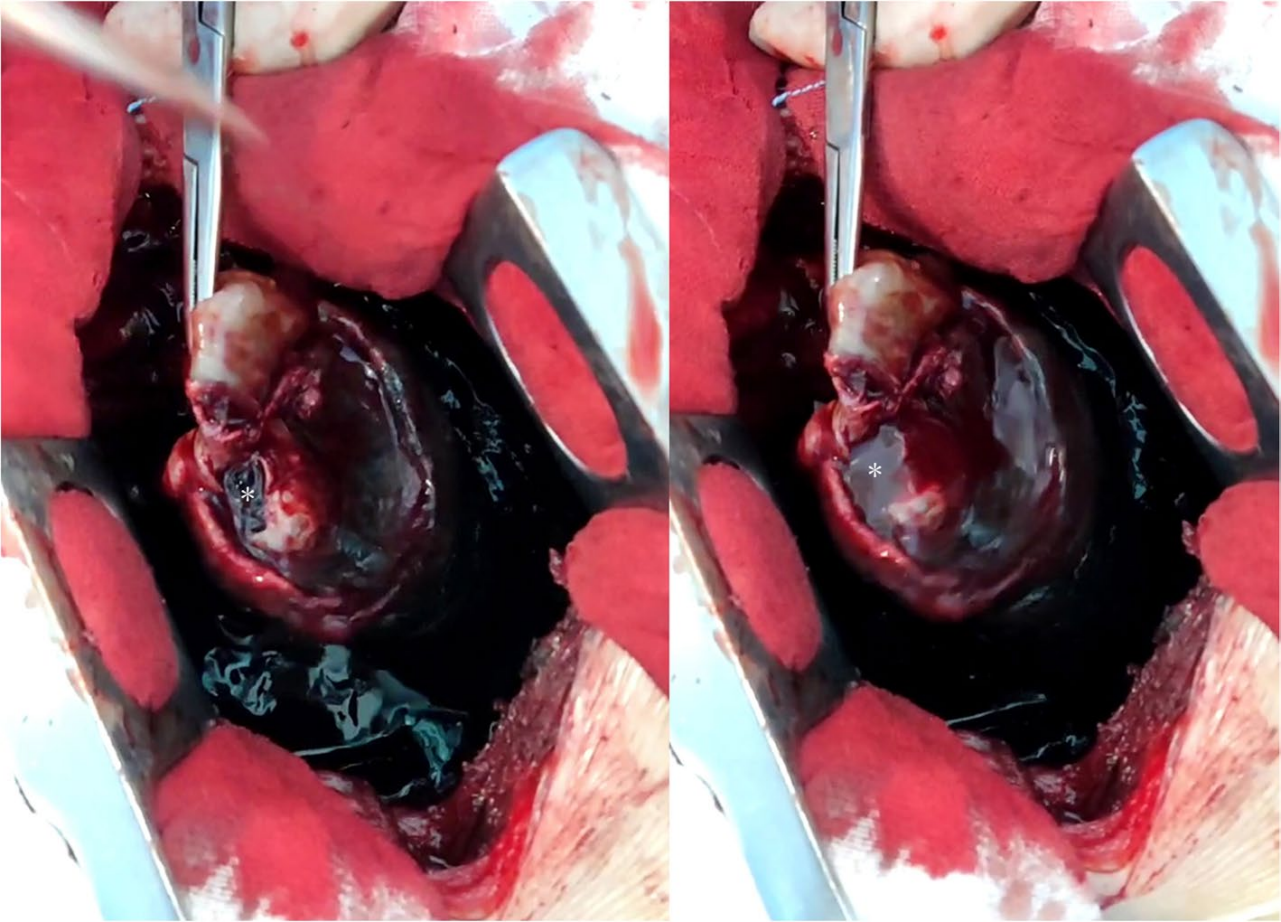
**A**-**B** Intra-operative findings demonstrated the ruptured site of PAVM with pulsatile bleeding during the cardiac cycle

After the procedure, the patient had intermittent fever and a slow drop in hemoglobin. Follow-up contrast-enhanced CT showed persistence of left hemothorax and remnant of arteriovenous fistula in the delayed phase (Fig. 4A-B) although feeding pulmonary artery was completely occluded. In order to avoid possible catastrophe from thoracic drainage according to data above, an urgent open thoracotomy was performed and this confirmed the existence of PAVM rupture. The diameter of the draining vein was approximately 12 mm and there was pulsatile bleeding at the ruptured site of PAVM with cardiac cycle (Fig. 5A-B). Then, the left lower lobe was successfully excised. In addition to the treated PAVM of the left lower lobe, another small PAVM in the right upper lobe was screened and remains untreated. No any abnormality was detected on cranial pre or post-embolization MRI, ruling out congenital intracranial arteriovenous malformation and ectopic infarction after interventional management. The patient’s vital signs were persistently stable after open surgery, and was discharged uneventfully 10 days afterwards. The results of the blood whole-exome sequencing revealed a pathogenic heterozygous splice site variant (c.511C > T) in the endoglin gene, an evidence of hereditary hemorrhagic telangiectasia type 1 (HHT1). Due to his economic limits, other relatives did not undergo the same genetic testing although we strongly recommended. On the follow-up at 3 months, 6 months, 12 months, and 18 months after the surgery, the patient was in good condition, with no signs of any bleeding symptoms.

## Literature review

We conducted a systematic search of the PubMed database for case report regarding PAVM complicated with hemothorax that had been published between January 2000 to July 2023. Only studies involving humans with full text were enrolled. A total of 30 articles were analysed to extract key information about radiographic diagnosis and treatment strategies of this disease entity. Thirty-one cases of PAVM accompanied by hemothorax including our case were summarily listed in the Table 1. The lesions in all 31 patients were located nearby the pleura.

**Table 1:** Summary of cases of pulmonary arteriovenous malformation associated with hemothorax

Case	Author/year	Age/sex	History	HHT	Symptoms	Diagnostic methods	Radiology findings				
							Rupture site	Size of PAVM	Pleural effusion	Other findings	Treatment
1	Melissa van den Bulck/2022 [6]	25/F	Supraventricular tachycardia, hypothyroidism	Unknow	Sudden onset left chest pain	CT, CTA	Left lower lobe	Unknow	√	Underlying lung collapse	Transcatheter embolization
2	Taylor J.Robinson/2022 [7]	34/F	Hypertension, infertility	√	Abdominal pain in the left upper quadrant/left back	CTA	Left lower lobe	Unknow	√	None	Transcatheter embolization
3	Obteene Azimi-Ghomi/2021 [8]	76/F	Hypothyroidism, trauma	Unknow	Chest pain and shortness of breath	Thoracentesis, CTA	Right middle lobe	1.8 cm	√	None	Lobectomy after failing to embolize due to feeding vessel tortuosity
4	Suxuan Liu/2021 [9]	17/F	None	Unknow	Sudden onset of dyspnea	CTA	Left lower lobe	Unknow	√	None	Transcatheter embolization
5	Jun Naito/2020 [10]	34/F	Epistaxis, telangiectasia of the tongue	√	Right chest pain	Thoracentesis, CT	Right middle lobe	Unknow	√	None	Lobectomy
6	Suguru Mitsui/2020 [11]	14/F	None	√	Left-sided chest pain and dyspnea	CTA	Left lower lobe	Unknow	√	None	Lobectomy via VATS
7	Pushpinder S Khera/2020 [12]	28/M	Epistaxis	Unknow	Right‑sided chest pain and breathlessness	Thoracentesis, CT, CTA	Right upper lobe	Unknow	√	Lung collapse	Transcatheter embolization
8	Jian Li/2019 [13]	51/M	Unknow	Unknow	Severe right chest pain	CT, CTA	Right lower lobe	4.5 cm	√	None	Lobectomy
9	Federica Di Guardo/2019 [14]	32/F	None	Unknow	Dyspnea and chest pain	Thoracentesis, CTA	Left lower lobe	4.0 cm	√	None	Lobectomy
10	Hu-Lin Christina Wang/2018 [15]	32/F	None	Unknow	Sudden -onset dyspnea and backache	Thoracentesis, CTA	Right lower and middle lobes	Unknow	√	Mediastinal shift	Lobectomy via VATS
11	Maja Crkvenac/2018 [5]	45/F	None	None	Chest pain and breathlessness	Thoracentesis, CTA	Left upper lobe	1.7 cm	√	None	Transcatheter embolization
12	Sze Shyang Kho/2018 [16]	45/M	None	None	Insidious onset of exertional dyspnea and left pleuritic chest pain	Thoracentesis, CTA	Left lower lobe	Unknow	√	Lung collapse	Transcatheter embolization
13	Oscar Rivero Rapalino/2018 [17]	34/F	None	Unknow	Tachypnea and hypoxia	CTA	Right lower lobe	3.5 cm × 3.1 cm	√	None	Transcatheter embolization
14	Hong -Wei Shang/2017 [18]	47/F	Cholelithiasis	Unknow	Massive hemoptysis	CT	Right lower lobe	3.0 cm × 2.5 cm	√	Atelectasis	Lobectomy
15	Mostafa El Hajjam/2017 [19]	57/F	Epistaxis, telangiectasia	√	Severe chest pain and acute dyspnea	CTA	Left lung	Unknow	√	None	Transcatheter embolization
16	Manohar Lal Gupta/2015 [20]	38/M	None	None	Progressive chest pain and breathlessness	Thoracentesis, CTA	Right lower lobe	2.6 cm × 1.4 cm	√	Mediastinal shift	Lobectomy via VATS
17	Feng Lin/2015 [21]	26/F	None	Unknow	Progressive dizziness and dyspnea	Thoracentesis, CTA	Left lower lobe	Unknow	√	None	Lobectomy
18	Vincenzo Di Crescenzo/2015 [22]	19/F	None	None	Dyspnea, hypoxia and leftsided pleuritic chest pain	CTA	Left lower lobe	3.0 cm	√	Compressive atelectasis	Lobectomy after transcatheter embolization
19	A A Khan/2015 [23]	71/F	Bilateral ankle oedema	Unknow	Dyspnea, right pleuritic chest pain	CT	Right middle and lower lobes	Unknow	√	Mediastinal shift	Lung repair surgery after transcatheter embolization
20	T. Dégot/2012 [24]	35/F	Cerebrovascular disease	√	Subacute dyspnea and left thoracic pain	Thoracentesis, CTA	Left lower lobe	Unknow	√	None	Transcatheter embolization
21	José Carlos López/2012 [3]	72/F	Rheumatic fever, mitral stenosis	None	Asymptomatic	Autopsy	Right lower lobe	6.5 cm × 5.0 cm	√	None	Death
22^a^	Nidhi Sood/2011 [25]	25/F	Gestational thrombocytopenia	Unknow	Progressive dyspnea and right -sided pleuritic chest pain	CT, CTA	Right lower lobe	1.0 cm	√	Compressive atelectasis	Transcatheter embolization
23^a^	Yinghao Zhao/2010 [26]	34/F	None	Unknow	Right -sided chest pain and dyspnea	CTA	Right lower lobe	Unknow	√	Mediastinal shift	Lobectomy
24^a^	Ira R. A Goldsmith/2010 [27]	48/M	None	Unknow	Sudden onset of left-sided chest pain and signs of shock	CTA	Left lower lobe	Unknow	√	Mediastinal shift	Lobectomy
25^a^	Adam M. Berg/2010 [28]	57/F	None	√	Chest pain	CTA	Right lower lobe	Unknow	√	Mediastinal shift	Transcatheter embolization
26^a^	Adam M. Berg/2010 [28]	64/M	Epistaxis, telangiectasia	√	Pleuritic chest pain and dyspnea	CTA	Right lower lobe	Unknow	√	None	Transcatheter embolization
27^a^	Takaki Ishikawa/2009 [4]	44/F	Epistaxis	√	Dyspnea and pain in the back	Autopsy	Right upper lobe	2.0 cm × 2.0 cm	√	Mediastinal shift	Sudden death
28	Muza ffer Elmali/2008 [29]	51/F	Telangiectasia, hemoptysis	√	Sudden onset chest pain	CTA	Right middle and lower lobes	3.0 cm	√	None	Transcatheter embolization
29	A. S. W.WONG/2004 [30]	33/F	Benign lung lesion	Unknow	Progressive dyspnea associated with productive cough	CT	Left lower lobe	Unknow	√	Mediastinal shift	Lobectomy via VATS
30	Pierre -Yves Litzler/2003 [31]	35/F	Epistaxis, telangiectasia	√	Sudden left thoracic pain and dyspnea	CT, CTA	Left lower lobe	Unknow	√	None	VATS to remove blood clots after transcatheter embolization
31	Present case	55/M	Epistaxis, intracavitary germ cell tumor	√	Sudden left chest pain and dyspnea	Thoracentesis, CT, CTA	Left lower lobe	3.9 cm × 3.1 cm	√	Passive atelectasis	Lobectomy after transcatheter embolization

*CT* Computed Tomography, *CTA* Computed Tomography Angiography, *F* Female, *M* Male, *PAVM* Pulmonary Arteriovenous Malformation, *HHT* Hereditary Hemorrhagic Telangiectasia, *VATS* video -assisted thoracoscopic surgery

^a^Case 22 and 27 experienced sudden death and autopsy revealed the presence of PAVM

Thirty-one cases included 24 females and 7 males. Most patients, including our case, presented with chest pain and dyspnea. Three cases complained of back pain, one case suffered from hemoptysis, and one case was asymptomatic. Out of the 31 cases, 29 cases were diagnosed through CT or CTA, mainly through CTA. The remaining 2 cases were diagnosed through autopsy.

Among the 31 patients, 26 cases occurred in the lower lobe of the lung, with 14 cases in the left lower lobe and 12 cases in the right lower lobe. Compared to the cases with explicitly measured lesion sizes among the 31 cases, our case exhibited a relatively larger PAVM with aneurysm, measuring approximately 3.9 cm × 3.1 cm. In the 16 cases that had completed genetic testing, 11 cases were definitely diagnosed with HHT, while 5 cases did not demonstrate relevant genetic mutation. Additionally, blood genetic testing was not conducted in 15 cases. Among the 11 patients with HHT, 7 cases had an obvious history of nosebleeds or telangiectasias.

Interventional embolization alone was utilized in 14 cases, surgery in 11 cases, and 4 cases undergo surgery after transcatheter embolization. There were 2 cases of sudden death unable to seek medical care timely. Additionally, surgical treatment was generally preferred for PAVM larger than 3.0 cm. Both cases of sudden death were attributed to intrapleural rupture of PAVM, and this further promised the potential serious consequences of PAVM combined with hemothorax. After a detailed review of the other 3 cases where embolization was followed by surgical management, it was identified that 2 cases were similar to ours. Initial embolization attempt failed, and subsequent surgery was chosen as a compensatory treatment. The reasons for embolization failure were attributed to the larger diameter of the feeding artery, the tortuous course of blood vessels, or the presence of multiple PAVMs associated with larger diameter feeding arteries and multiple feeding arteries. In another case, the patient had a long-standing significant hemothorax, attributed to the existence of PAVM. After undergoing interventional embolization for the PAVM in the left basal segment, the left lung still failed to re-expand. Although four patients above initially underwent embolization treatment or DSA, surgery was ultimately required to achieve the complete cure or facilitate fast recovery.

## Discussion

Pulmonary arteriovenous fistula is a rare PAVM that is caused by a disorder in the formation of blood vessel septa in the pulmonary vascular plexus, resulting in underdeveloped or degenerated capillaries and a direct connection between the pulmonary artery and vein, which leads to an intrapulmonary right-to-left shunt. It is more prevalent in females than that in males, and the majority of affected regions are located in the lower lobe. PAVM can be classified as congenital or acquired according to its cause. Congenital PAVM is often associated with hereditary hemorrhagic telangiectasia (HHT), while acquired PAVM is often caused by factors such as liver cirrhosis, schistosomiasis, infection and trauma. Approximately 70% of PAVM is associated with HHT, which is a rare autosomal dominant inherited disorder [16]. HHT often manifests as multiple arteriovenous malformations in the skin, mucous membranes, and internal organs. The malformed blood vessels in HHT can also be tortuous and coiled, or even appear tumor-like [32]. Familiarity with the multi-system involvement in this disease can help in searching for diagnostic clues and related evidence. Our patient had a previous history of epistaxis, suggesting involvement of the nasal mucosa. Combined with skin telangiectasia, multiple PAVMs and genetic testing result, these data allowed us to make a confidential diagnosis of HHT.

Clinical symptoms of PAVM depend on the degree of right-to-left shunting. When the right-to-left shunt exceeds 20% of total systemic circulation, the patient may exhibit cyanosis, clubbing of fingers and toes, shortness of breath, and recurring systemic embolization, but the most significant manifestation is asymptomatic hypoxemia [1, 32]. PAVM can also rupture and penetrate into the lung parenchyma, bronchial trees, and pleural space, leading to corresponding symptoms such as hemoptysis, pulmonary hemorrhage, dyspnea, chest pain, and hemothorax [1, 5, 33, 34].

As described in our case, the PAVM was located in the peripheral lung tissue and adjacent to the pleura, which caused massive hemothorax after its rupture. The reason for this rupture may be that the patient’s emotional excitement led to an increase in pulmonary circulation and an abrupt fluctuation in intrathoracic pressure, which increased the force of blood flow on the wall of PAVM and caused the weak part of PAVM to rupture and bleed, as described in the literature [35].

Contrast-enhanced computed tomography remains the gold standard for depicting the anatomy of PAVM. It not only allows for the detection of feeding arteries and draining veins, but also the assessment of whether there is a pulmonary aneurysm rupture [5, 32, 36]. When there is hemothorax or hemoptysis in those patients with PAVM, the presence of the “anomalous bulge” sign on CTA and the “double shadow” sign on DSA usually represents for the rupture of a PAVM [37]. Among the 29 cases with radiological data, 18 cases exhibited the aforementioned features, which were extremely helpful in confirming the diagnosis. DSA can further confirm the diagnosis, and embolization treatment can be simultaneously completed in almost patients with PAVM. Among our retrospective 18 cases, only one cases failed to perform embolization because of tortuous pulmonary artery. With the advancements in modern medical technology and the advent of multi-detector CT, CTA has already replaced DSA as the commonest diagnostic method. According to our study of 31 cases, except 2 patients who suffered sudden death, the remaining 29 cases were diagnosed using CT or CTA before management.

Once a diagnosis of PAVM has been established, based on its potential complication and gradual growth, it is theoretically advisable to treat for all symptomatic patients and any asymptomatic patient with one or more PAVM with a feeding artery diameter of more or equal to 2-3 mm [5]. The treatment aims to prevent the continuous growth or hemorrhagic complication from rupture. Treatment modalities mainly include interventional embolization and surgery. PAVMs are historically treated with open resection, and in recent years video-assisted thoracoscopic surgery for PAVM has become popular. With the advancement of endovascular techniques, embolization has become one of the mainstay for treating PAVM. Compared to surgical resection, embolization technique offers less invasiveness and lower risks as well as preserving more healthy lung tissue [38]. In the manipulation of embolization treatment, vascular access is obtained through catheter insertion, and the blood supply artery is selectively occluded to eliminate blood flow through the PAVM. For small draining vein cases of most PAVMs, regardless of ruptured complication, interventional embolization can usually reach desirable outcome in clinical practice. Among our investigated cases, 14 out of 18 patients achieve successful outcomes with simple embolization as the main therapy. The common characteristic among these patients is the presence of simple PAVM with small lesion size and small draining vein diameter. However, this method may have some potential shortcomings such as incomplete embolization, vascular recanalization, formation of collateral blood vessels, iatrogenic embolus dropout, ectopic embolization, and persistent pulmonary arteriovenous fistula with incomplete treatment and easy recurrence [39]. For patients with unsuccessful embolization, surgical resection may be an alternative treatment. Simultaneously, surgery can also evacuate any accumulated pleural fluid or blood clots within the thoracic cavity. One of the cases in our review involved performing video-assisted thoracoscopic surgery immediately after a successful embolization therapy so as to remove residual clots and pleural effusion. Since no active bleeding was observed intraoperatively, further lung lobectomy was unnecessary [31]. So far, the standard treatment in the setting of PAVM with hemothorax remains to be further elucidated.

Combining systematic review of literature cases and our patient, the important factors related to the failure of catheter-based treatment were the presence of a large and tortuous drainage vein. The detailed manifestations include: the patient’s symptoms of chest pain and dyspnea persisted or even worsened after catheter treatment; the value of hemoglobin showed a gradual decrease, which suggested the existence of active bleeding; there was no evidence of a decrease in the amount of pleural effusion compared to previous CT scan. Repeated enhanced CT scans after PAVM embolization showed that the aneurysm did not shrink remarkably. These findings above strongly suggest that the catheter-based treatment was unable to effectively control active bleeding caused by PAVM rupture, so alternative treatment should be considered. Therefore, further exploratory surgery was necessary in our case. During operation, it was found that the feeding artery was totally occluded, but the ruptured orifice of PAVM was still intact. It was bridged to the left atrium by a relatively large drainage vein. With the systolic and diastolic beat of the heart, the backflow of blood again entered the ruptured aneurysm cavity in a tidal manner. Finally, the left lower lobe was removed with an excellent outcome.

## Conclusion

Through a retrospective analysis of diagnostic and therapeutic experiences from our case, in the context of PAVM complicated with hemothorax, especially in those cases where there is a relatively large drainage vein and the patient’s stable hemodynamics, radical surgical treatment is strongly recommended.

## Data Availability

We declared that materials described in the manuscript, including all relevant raw data, will be freely available to any scientist wishing to use them for noncommercial purposes, without breaching participant confidentiality.
